# Flying under the radar: a qualitative examination of self-diagnosed ADHD and identity formation in young women

**DOI:** 10.1186/s40359-026-05732-7

**Published:** 2026-10-05

**Authors:** Matilda A. Frick, Cecilia Cederlid, Linn Hegdal, Jan Grimell

**Affiliations:** 1 Department of Psychology, Stockholm University, Stockholm, Sweden; 2 Department of Medical Sciences, Child and Adolescent Psychiatry Unit, Uppsala University, Uppsala, Sweden; 3 Department of Sociology, Umeå University, Umeå, Sweden

**Keywords:** Self-diagnosed ADHD, Identity, Young women, Qualitative research, Thematic analysis

## Abstract

**Background:**

In recent years, the prevalence of attention-deficit/hyperactivity disorder (ADHD) diagnoses has increased sharply, in many western countries, including Sweden, especially among women and adults. Concurrently, more people seem to identify with ADHD without having a formal diagnosis, a phenomenon that has been highlighted in the media but not examined thoroughly in research. As women with ADHD are diagnosed later in life compared to men, self-diagnosis in young women may be an important piece of puzzle to understand diagnostic processes and identification. The aim of the current study was to improve the understanding of the motives and processes underlying self-diagnosed ADHD in young women, with a special focus on identity formation.

**Methods:**

Semi-structured interviews were conducted in 2025 with 15 women aged 18–25 years with self-diagnosed ADHD and self-rated ADHD-symptom levels above the cut-off for probable diagnosis. The sample was recruited via Meta and the interviews were analyzed using thematic inductive method.

**Results:**

The analysis generated six themes. Three themes related to how young women describe their self-diagnosed ADHD; *recognition in ADHD symptoms*,* experience of invisibility and a lonely struggle*, and *ambivalence towards assessment and diagnosis.* An additional three themes to how they articulate their self-perception; *managing demands and expectations*,* difficulties related to social roles and relationships*, and *feeling different and seeking acceptance.*

**Conclusions:**

Self-diagnosis played an important role in identity development by validating difficult experiences for young women with ADHD-like symptoms. Strives to live up to being a “good girl”, including high academic achievement, disguised their experienced difficulties and gendered norms may contribute to self-diagnosis and medicalization. Their invisible struggles had a negative impact on self-image, and they experienced being left to fend for themselves in dealing with the demands of society.

**Supplementary Information:**

The online version contains supplementary material available at https://doi.org/10.1186/s40359-026-05732-7.

Over the last decades, the prevalence of attention-deficit/hyperactivity disorder (ADHD) has increased sharply in many western countries, such as the US, Canada, Australia, Germany, and the Nordic counties including Sweden with a pronounced increase among adult women [1–4]. In tandem, self-diagnosed ADHD, that is identifying with the condition in absence of a formal diagnosis, also seems to have increased [5]. Self-diagnosed ADHD is widely recognized in traditional and social media but so far relatively unexplored scientifically [6, 7]. Thus, in the current study we set out to explore the motives and processes underlying self-diagnosed ADHD in young women.

ADHD is a developmental condition characterized by enduring difficulties stemming from inattention and/or hyperactivity/impulsivity, evolving in childhood and often persisting into adulthood [8]. ADHD entails an increased risk of academic difficulties and psychiatric comorbidities, including anxiety and depression [9]. Recommended interventions are psychoeducation, cognitive aids, as well as psychological and medical treatment [10]. Globally, the estimated prevalence is 5–7% of children/adolescents and 2–3% of adults [11]. However, national prevalence’s vary widely [12] and increases in diagnoses does not necessarily reflect an increase in the underlying ADHD phenotype [13]. The reasons for the increase are not fully understood, but are likely due to a combination of factors such as increased awareness [14], reduced stigma [15, 16], a more complex society, and increased educational demands [17].

Receiving a diagnosis can be an important piece of puzzle in individuals’ identity formation and provide an explanatory framework for difficulties in every-day life [18, 19]. Similarly, a self-diagnosis may serve as a validation of difficult experiences and seems to be underpinned by multifaceted reasons [20]. For instance, a self-diagnosis may be motivated by a search for identity and belonging [21, 22]. Social media and informal health-related online communities appear to constitute influential sources of information for self-diagnosis [21, 23]. Potential negative consequences of self-diagnosis include incorrect diagnosis [5] and lack of treatment [24], accompanied with personal suffering [25] and economic costs for the person and society [26, 27].

Arnett [28] refers to the period of development for people aged 18–25 years as *emerging adulthood*, a period of transition from adolescence to adulthood characterized by identity building and integration. Many women report difficulties with living up to the demands and norms in society [29]. This may contribute to self-diagnosis and medicalization, that is a tendency in society to increasingly interpret and treat every day experiences, emotional states, and behaviors as medical problems [30]. As such, there is a risk that typical human experiences are pathologized, and that underlying societal problems will be defined as health problems [31]. Furthermore, Broeker and Arnaud [5] point to structural factors related to time and cost as causes of self-diagnosis. Often, there are long waiting lines for ADHD assessments and paying for an assessment in private care is not possible for everyone. Therefore, self-diagnosis may reflect a current underdiagnosis that in the long-term may lead the individual to seek a formal assessment [32]. Further, as women might display a different symptom profile than men, their difficulties may not be recognized [33], and they therefore risk to be left to fend for themselves by self-diagnosing. Finally, at the societal level self-diagnoses ultimately risk leading to overdiagnosis by watering down diagnoses, as the threshold for symptoms is lowered and the meaning of the diagnosis changes [34]. Overall, this highlights the importance of a better understanding of the phenomenon of self-diagnosed ADHD and its underlying motives, processes, and consequences.

The current study aims to investigate the motives and processes underlying self-diagnosed ADHD in young women, by addressing the following research questions: How do young women describe their self-diagnosed ADHD? How do young women with self-diagnosed ADHD describe their view of themselves?

## Method

A qualitative, interview-based approach was deemed appropriate for this study, as it enables the exploration of participants’ subjective experiences regarding the motives and processes that had led to their self-diagnosis of ADHD. Employing an in-depth qualitative interview method can provide insight into how participants conceptualize and reason about the phenomenon under investigation [35]. The study was funded by The Foundation Sunnerdahl’s handikappfond (F8/23), conducted in accordance with the Declaration of Helsinki, and ethically approved by the Swedish Ethical Review Board (Dnr. 2023-06162-01).

### Procedure, materials, and participants

The participants were recruited via advertisements on Meta (published on Facebook and Instagram) in January and February 2025, directed to women aged 18–25 years living in the greater Stockholm area. Recruitment via Meta was chosen as approximately 90% of young women in Sweden use social media [36]. To be included, participants had to be biologically born women aged 18–25 years, be self-diagnosed for at least one year, and display self-rated ADHD symptom levels above the cut-off for probable diagnosis. Exclusion criteria were formal ADHD diagnosis, autism, psychotic disorder, intellectual disability, being in the queue for an ADHD assessment, having previously undergone an ADHD assessment, and high symptom levels of depression, high alcohol consumption, or use of illegal drugs.

Interested individuals filled out a questionnaire regarding inclusion and exclusion criteria, age, sex, sociodemographic information, and symptoms of ADHD, depression, alcohol use, and substance use. The Adult ADHD Self-Report Scale Adolescent version (ASRS-A) [37] was used to estimate the participants’ ADHD symptoms. For depression, we used the Montgomery Åsberg Depression Rating Scale (MADRS) [38]. To measure alcohol use, we used the Alcohol Use Disorders Identification Test (AUDIT) [39] and The Drug Use Disorders Identification Test (DUDIT) [40] was used to identify drug use.

A total of 55 people registered their interest in participating. Grounds for exclusion were previous assessment for ADHD (*n* = 11), in queue for ADHD assessment (*n* = 4), formal ADHD diagnosis (*n* = 3), formal autism diagnosis (*n* = 8), formal emotionally unstable personality disorder (*n* = 1), indication of severe depression (*n* = 1), indication of alcohol abuse (*n* = 8), and living elsewhere (*n* = 7). Twenty qualified for participation, of which 17 were contacted and booked for interview. Two were prevented from participating due to time constraints, resulting in 15 included participants. As a token of appreciation, the participants received a gift card worth SEK 100.

See Table 1 for participant characteristics. All participants had self-rated ADHD symptoms, clearly above the proposed threshold of 30 points that indicates an increased likelihood of ADHD [41]. Their ratings of depressive symptoms ranged from no to moderate depression. Regarding alcohol use, eight participants were below the limit for risk use whereas seven participants were just around the cut-off value and were admitted to the study after individual assessment of their score. None of the participants had an active or previous drug use.

**Table 1:** Participant descriptives

Measure	Range (M; SD)
Age	18–25 years (22; 2)
Lenght of self diagnosis	1–15 years (6; 3)
ADHD symptoms^1^	39–66 (51.20; 7.82)
Depressive symptoms^2^	6–25 (17.07; 5.31)
Alcohol use^3^	0–10 (4.60; 3.70)
Drug use^4^	0

Note. The following scales were used: ^1^The Adult ADHD Self-Report Scale Adolescent version, ^2^the Montgomery Åsberg Depression Rating Scale, ^3^the Alcohol Use Disorders Identification Test, and ^4^The Drug Use Disorders Identification Test

Participants had been self-diagnosed for a mean of 6 years. Ten participants were born in Sweden, three in other European countries, and two in Asia. All participants lived in the greater Stockholm area and studied (*n* = 14) or worked (*n* = 1) as their main occupation. Five participants had previously been diagnosed with generalized anxiety disorder, obsessive-compulsive disorder, dyslexia, or depression. None receive treatment for their self-diagnosed ADHD.

Semi-structured interviews were conducted based on an interview guide (Supplementary material 1), developed by the authors and adapted from a previous guide used with diagnosed individuals [15, 18]. Open-ended questions with in-depth follow-up questions were asked related to being a woman, identity, self-diagnosed ADHD, and the impact of perceived symptoms on studies, work, and relationships. Examples of questions were “How come you think that you might have ADHD?” and “Do you feel that the fact that you are a woman has affected how it is and has been for you to live with ADHD-like symptoms?”. After conducting trial interviews, CC and LH, who were trained in qualitative interview methodology, conducted seven and eight interviews each. All interviews took place at Stockholm University and took about an hour. Before the interview, all participants provided oral and written informed consent to participate. The interviews were audio recorded and transcribed with the AI tool Amberscript (www.amberscript.com). The transcriptions were pseudonymized and corrected manually. Saturation was assessed by estimation that additional interviews no longer generated new insights or themes in response to the research questions [42].

### Analysis

Thematic analysis was applied using the six steps formulated by Braun and Clarke [43]. The analysis was inductive, in that themes were based on patterns in the data material and not on existing theory. The analysis was carried out by the second and third author under supervision of the first author:

1. Familiarization with the full data by reading through the transcribed interviews.
2. Initial codes were created (see Table 1 for examples). The first two interviews were coded jointly, and then the coders coded their own interviews individually. Every third interview was coded by both coders to increase interrater reliability. Segments that were perceived as relevant in relation to the research questions were coded. Eleven of the total 15 interviews were coded first, the remaining four were saved for saturation analysis.
3. About 1500 codes were entered into an Excel document. The coders discussed potential groupings based on the codes, which generated eleven initial themes (see Table 1 for examples).
4. Selected themes were revised and condensed into six. Hard-to-place codes were discussed and repositioned. Here, a saturation analysis was carried out with the four remaining interviews. The saturation analysis did not generate any new themes; all codes could be subordinated to already existing themes.
5. The content of each theme with related codes was reviewed to clearly define the respective content. Each theme was given its final label.
6. Final themes were compiled under the results section. Each description contained selected quotes to reinforce the essence of each theme.

## Results

**Fig. 1:**
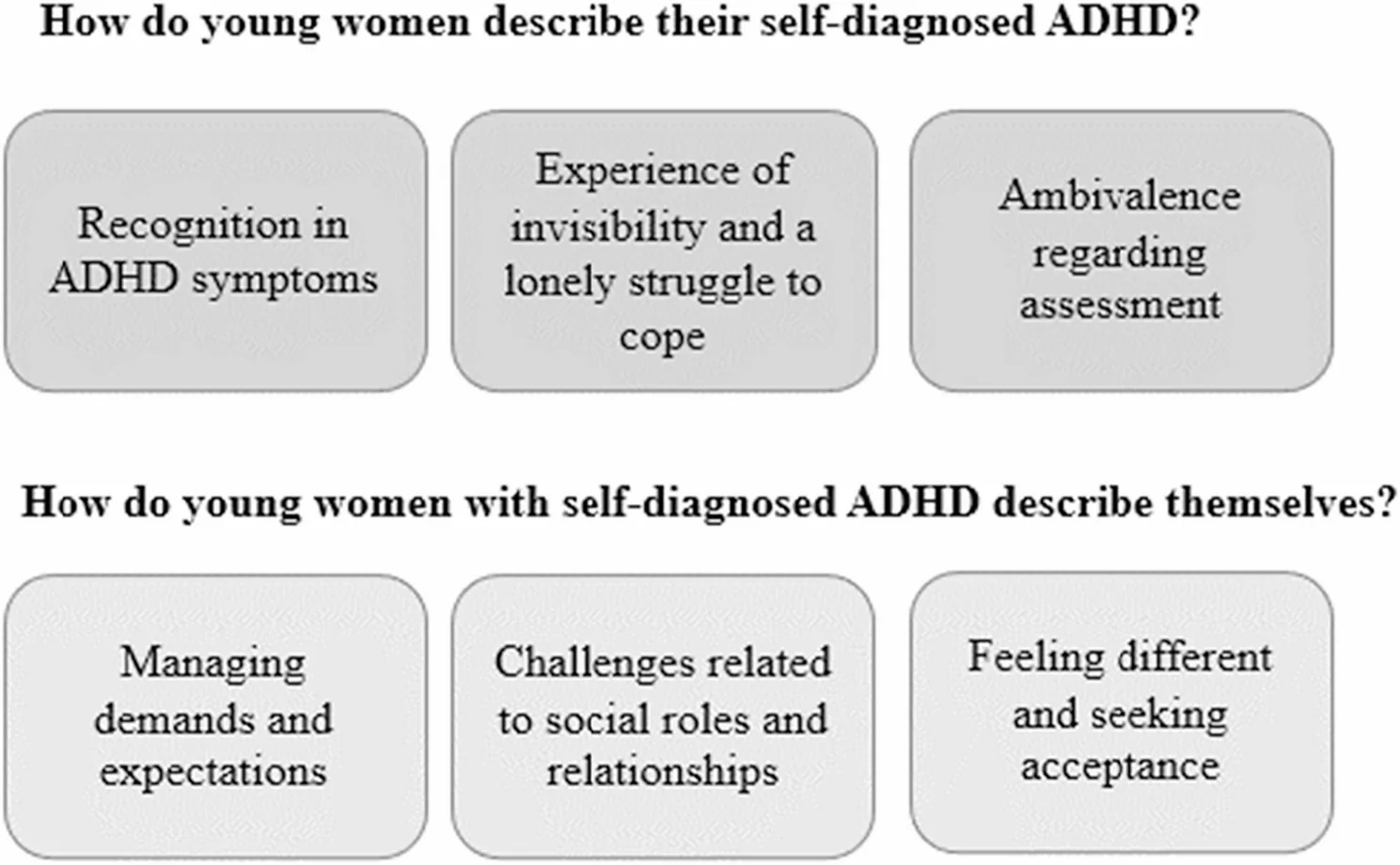
Depiction of the research questions and obtained themes

The analysis yielded six themes (see Fig. 1); three relating to how young women describe their self-diagnosed ADHD and three to how young women with self-diagnosed ADHD articulate their self-perception. To protect anonymity, participants are referred to by fictitious names (Table 2).

**Table 2:** Examples of codes and themes from some of the interviews

Interview	Code	Theme
Inez	Always difficult to initiate	Recognition in ADHD symptoms
Eve	Stigma around diagnosis	Ambivalence about assessment and diagnosis
Clara	Feeling of being misunderstood and not fitting in	Feeling different and seeking acceptance

Note. The names of the participants are fictitious

### How do young women describe their self-diagnosed ADHD?

Here, three themes emerged that describe how the participants came to recognize themselves in the ADHD diagnosis, their experience of having to deal with their difficulties on their own, and thoughts about the proposed advantages and disadvantages of a formal diagnosis.

#### Recognition in ADHD symptoms

A central theme concerned how the participants recognized themselves in the ADHD symptoms, how these manifested in different parts of their lives and caused problems.

The participants had started to think about aspects of their functioning as ADHD in different ways. Lisa stated that at some point during high school, she found an earlier evaluation where a teacher described that she often daydreamed and had difficulty focusing. Lisa, who had good grades in elementary school but struggled in high school, realized that she had always had difficulty with inattention and the thought of ADHD came up. In contrast to Lisa, others described a process where several factors contributed to the suspicion of ADHD. For instance, the participants had recognized themselves in traits of family members or friends diagnosed with ADHD, and close ones had pointed out difficulties. Some had sought information about ADHD on social media and done self-tests online. The fact that family members had similar traits was mentioned as a validation for the self-diagnosis.

The participants described symptoms of inattention, especially difficulties in maintaining focus on tasks and conversations. “*I sit down and try to learn. Maybe a quarter of an hour passes? And then I do something else. Or I get distracted*,* I look at my phone. I’m leaving*,* who’s going to stop me?*”, Clara stated about her studies. The participants described getting distracted or stuck in their own thoughts. Inez said that she starts cleaning but doesn’t finish because she constantly finds things that distract her.

Controlling one’s motivation and drive appeared to be difficult. For some, it was easier to concentrate if something was interesting and most said that they were able to hyper focus. “*I can sit for a whole day and just do something that maybe my friends don’t understand: *‘*How can you sit and crochet a whole laundry basket over a whole day and forget to eat?*’” said Konstantina. Lisa described: “*We had an exam*,* there were ten writing questions and I remember that I got so bored halfway through*,* that I just wrote pretty badly on the rest because I just wanted to get out*.”

The participants expressed various difficulties related to organization, planning and time management. “*It could be that I have a lecture at three o’clock and then I wake up at eight and then I have the whole day ahead of me and then I can’t get it together*,” Anna explained. Like Olga, most stated that it is difficult to get started: “*Even if I want to*,* it doesn’t work. If we were to be given an assignment during class time. I would sit there for 10–15 min at least. How do I get started with this?*”. Most described that they postpone tasks until the last minute, which leads to stress and pressure. “*I feel like I have to wait for some magical moment for me to get started*,” Mona said about cleaning and went on to say that it can also be difficult to finish things: “*if I clean*,* I have to clean really clean*,* and then it will also take several hours*.” The participants also brought up that they forget what needs to be done or lose things such as keys and mobile phones.

Furthermore, the participants described difficulties in regulating activity level. Most prominent was a constant mental activity: “*It feels like my brain is constantly running at full speed*,” said Anna, while Jamila expressed it as “*there are sirens and alarms and everything going on at the same time*” in her head.

Impulsive behavior was also described. They had done “*stupid things*”, said things they regretted, and had difficulty with impulse control related to money or alcohol. Anna had difficulty listening to others and often gets criticism from friends for this. The majority also experienced strong and difficult-to-control emotional reactions. Jamila said “*I have always felt that my feelings feel a bit like you have plugged in an amplifier*,* so if I get sad I get really sad*,* completely devastated*,* and if I get happy*,* it is almost euphoria*.”

The participants described how the ADHD-like symptoms caused problems for them related to studies, work, at home, and socially. Academic difficulties were the biggest problem, for some it had resulted in an accumulation of residual exams that delayed their education. The participants with work experience described of lack of motivation when work becomes boring, forgotten or delayed tasks, and colleagues who had to cover up for them. Difficulties in planning had caused irritation among friends, and Gina expressed concern about how social relationships are affected by her saying ill-considered things.

While Lisa did not link her self-diagnosed ADHD to anything positive at all, most participants could see some positive aspect, such as the ability to hyper focus, to chat with anyone, to be creative and resourceful, and that friends appreciate one’s energy and spontaneity. “*There are some days when the motivation is at its peak and you just pour through life and you have so much energy and you are so happy. And you talk to people right and left and are generally bubbly*,” said Gina.

#### Experience of invisibility and a lonely struggle to cope

The participants described that their difficulties had been invisible to the surroundings to varying degrees and that they had to cope with their difficulties on their own. Gina described her experience of being self-diagnosed as “*then maybe there are people who fly a little under the radar. … And maybe I’m one of those who hasn’t been recognized*.”

One perception was that their symptoms had not been noticed because they did not fit into the stereotypical image of ADHD. The participants had more often had difficulty with inattention than with hyperactivity and rarely been disturbing at school. Konstantina said that she had obvious difficulties concentrating, which was not taken seriously because she was not troublesome. Instead, she was placed between the rowdy boys at school: “*then you sat in the middle and couldn’t focus because you were just a little quieter*.” Jamila said that she was perceived as calm at school even though it was “*screaming in her head*”. Most believed that a lack of understanding of ADHD in women has meant that they have not been seen.

In line with the experienced invisibility, they had made effort to hide or compensate for their difficulties. The majority stated that it had gone well in school, although some had to fight for it. Inez said that she always received top grades without having to study, while Daniela said that although she had received good grades in elementary school, she had stressed a lot. Their study performances seemed to have masked difficulties and some of them had been told by parents or healthcare contacts that they cannot have ADHD because they performed too well in school.

The participants described that they had developed strategies to meet the demands placed on them and that these strategies masked their difficulties. Daniela wrote detailed to-do lists and Eve always put important things in the same place and planned her entire day in detail, including travel time and meals. The participants also described strategies for social interaction, that masked difficulties. Clara has learned to cold talk through YouTube and Konstantina said that she had learned that you have to ask questions to preserve relationships.

Although the strategies made them appear ‘normal’, they came with a cost. They described that the studies often took longer and required more energy than for others. After a week of exams, Jamila blocked the weekend for recovery. They had tested strategies that did not work or that stopped working when the demands increased. Others expressed frustration at having tried everything but not found strategies that worked. Few stated that they had received support at school and Felicia commented on the lack of interventions: “*It’s a bit this*,* especially for girls and women*,* that it’s not as often diagnosed*,* precisely because it’s going pretty well anyway. So no*,* I haven’t gotten that*.”

The participants described that their parents had not seen or understood their difficulties and this was in some cases due to skepticism towards diagnoses. Konstantina had had several healthcare contacts during her childhood and experienced that the healthcare wanted to find other explanations for her difficulties, only one doctor has raised the issue of ADHD. “*I was told so much about my anxiety problems and so on that I had low self-esteem. And so it was. There were a lot of excuses*,” she said. The participants had had contact with health care, but related to other diagnoses, such as anxiety, depression, obsessive-compulsive disorder, eating disorders, stress, burnout, and trauma. Several participants said that school or health care at some point had raised suspicion of ADHD, but that for most it had led to nothing.

#### Ambivalence about assessment and diagnosis

The participants expressed ambivalence about a formal assessment and weighed the pros and cons of a possible diagnosis. Eve expressed that she was not interested in being assessed right now as she had made her everyday life work. However, she said that an assessment could be of interest in the future. Difficulties regarding having to deal with starting an assessment were expressed, partly due to perceived ADHD symptoms. A few years ago, Clara was given the opportunity to be assessed, all she had to do was to click on a link: “*I’m stuck in the fact that I’ll do it tomorrow. I’ve been for five years now. That link is in the email*,* but I haven’t clicked on it.*” Ambiguous thoughts seemed to be one reason why self-diagnosis was allowed to suffice. The participants also mentioned that long waiting-lines and money were obstacles to seeking an assessment.

Proposed benefits of a possible diagnosis were getting concrete tools to help with one’s difficulties and an increased understanding of oneself. Clara expressed: “*It would be nice to get on paper that I’m not like*,* that I’m not just stupid in the head. It would have been very nice that there is a reason why I have difficulty with things*.” If they met the criteria for diagnosis, family members, friends, and colleagues might gain a greater understanding of their everyday struggles.

Reasons for not moving forward with an assessment, were risk of stigma and getting an unwanted label that could limit you in working life. Further, some expressed uncertainty about whether they would meet the criteria for a diagnosis at all. Mona said that she was afraid of “*not having what it takes*” and expressed that “*I’m afraid that I’ll get there and that they’ll be like: *‘*Oh*,* another one who has self-diagnosed herself*,* she has no problems at all*.’” Daniela expressed thoughts that she may be functioning too well to be able to get a diagnosis: “*It feels like I’m functioning a little too well for that to work*… *what if I imagine some of this and it’s not really that bad.*”

Finally, the participants were ambivalent about using the label explicitly and described that they did not go around telling others that they think they have ADHD. Mona said: “*It’s such a common phenomenon in some way*,* that people self-diagnose. So it has become a very easy to find explanation for things that are difficult*,* I think*”, and that she did not want to use it as any kind of excuse. In all, self-diagnosis seemed to be something positive. Konstantina:

“*It’s not that I use the diagnosis as if I feel that I absolutely have it*,* but it’s more that I identify quite a lot with parts of it and then kind of find some peace in the fact that this might be the case. It doesn’t have to be that I have ADHD*,* but that I can relate a lot more and that’s in some way*,* it’s a little reassuring. So little acceptance*.”

### How do young women with self-diagnosed ADHD describe their view of themselves?

Here three themes emerged, that describe dealing with high demands from the environment and themselves, difficulties navigating social relationships, and the feeling of not fitting in.

#### Managing demands and expectations

The participants experienced high demands and a desire to constantly want to improve. A common perception was that the environment’s demands and expectations of women and men differ. For example, Jamila had been told several times that she was sensitive, something she did not believe a man with the same symptoms would have been told. Gina mentioned that women are expected to be soft in social contexts, which does not apply to men, and reflected on the fact that she herself can yell at people sometimes. She had always wished that she was better at school but did not perceive that her brothers had the same expectations to succeed academically.

The desire to be good and perform well in school was expressed throughout and seemed to be a central part of their self-image. On the other hand, increased academic demands meant difficulties and an ever-present pressure to perform. Going from being ‘the good girl’ who had an easy time at school to suddenly no longer getting their studies together was described by many as shocking. The transition to high school was described by Jamila as a “*jaw-dropper*” as she was no longer allowed to submit essays after the deadline. She could no longer get her studies together and said: “*I thought high school was really difficult. I still had good grades*,* but not as good as before*,* so it was some kind of identity crisis*.”

The expectations of being a good girl in combination with what they now perceived as ADHD symptoms meant great frustration. Thoughts such as “*why is it so easy for everyone else?*” could relate to both school achievement, friendships, and future goals. Anna stated: “*I don’t have to have the highest grades*,* but it still gets hard because it feels almost unfair in a way. Because I know I can*,* but I can’t. That is not possible in practice*.” Daniela wished she was more efficient academically because “*it takes a very long time to do something that I think should actually be much easier for someone else*.” Maintaining the image of oneself as driven and ambitious, while experiencing symptoms they now associated with ADHD, was difficult.

#### Challenges related to social roles and relationships

The participants had spent a lot of time trying to understand how social relationships are created, functions, and are maintained. The social difficulties seemed to have a negative impact on self-image and in many cases contributed to a need to take on different social roles, as a way of trying to fit into the community.

Daniela described a wish that social situations should be more natural: “*More effortless as well. That it’s easier and that you don’t*,* don’t freeze*.” Eve expressed that she did not know how to make friends and that she could feel fear of rejection and a worry about wasting other people’s time. As a strategy for dealing with social contexts and not revealing their experienced difficulties, the participants described observing how others acted.

The participants described that they had taken on different social roles depending on the context and that they needed to adapt in order to live up to the expectations of others. Jamila described a fear of being seen as different if she did not step into her different roles. When growing up, she had so many different social contexts and roles that it became “*chaos on birthdays*” when all the different groups of friends were gathered in the same room and knew completely different versions of her. She described stepping into a role as “*just showing a certain part of me and hiding the other part. And then you moved it*,* depending on who you were talking to*.” When asked how it works in new contexts, Mona replied: “*It’s going well*,* but I still think it’s very stressful. I want to be liked by everyone*,* and the slightest sign that someone wouldn’t like me I take very hard*.” Having several superficial acquaintances but few close friends was further described as part of the difficulties with relationships.

The participants described how perceived ADHD symptoms affected their relationships. Anna described that she had had a very difficult time with both close and superficial relationships because of her planning difficulties: “*It could be that we were going to meet*,* have a coffee and I just cancelled because something came up*”. Konstantina further described experiences of forgetting to contact friends and acquaintances if they are not in front of her. She said that this results in superficial relationships. She also said that it was easier to maintain relationships with childhood friends as it means a different kind of security: “*They know a little bit about what I am like. They know that I’m a bit scattered and I forget things*.” The security and acceptance of those who had been present for a large part of their lives seemed to be of great importance to the participants.

#### Feeling different and seeking acceptance

The participants consistently described a feeling of being different and not fitting in, without really understanding why. “*I remember or start to think about that feeling that you’re a little bit left out even though you weren’t anyway*,” Felicia expressed. The participants gave different descriptions of how this had affected their self-image. Some have carried a strong feeling that they are to blame and that they are not good enough.

The participants felt that sudden difficulties in school had affected their self-image and challenges in their studies had continued to eat away at their self-esteem. Clara said that she had thought about dropping out of university: “*because I felt that maybe I’m too stupid*,* I don’t know why everyone else thinks this is so easy. So it was absolutely that I felt bad for a very long time*.”

The participants had a feeling of inadequacy and an inner critical voice that said that they had to change in order to be accepted. “*I’ve felt so much throughout my life that I wish I were someone else*,” said Gina. The feeling of being different had resulted in a tug-of-war between the desire to be themselves and the desire to fit in. There was a concern about not being accepted if you are open about your difficulties and the participants reflected on how much they can or want to adapt to the expectations of society. Most stated that they had at least one relationship where they could be themselves. Some described how they have found a context where they feel accepted, such as a workplace, a study environment, leisure activities or friendships with like-minded people.

The search for a stable identity was common. For some, self-diagnosis was a step towards understanding and accepting themselves. “*I think first and foremost it’s a way for me to get to know myself and accept myself. … And it’s not something I need to get rid of*,* but I can accept and I can find strategies*,” said Jamila. The majority described that they had been able to accept themselves more easily with age and that they had begun to see their strengths and weaknesses as part of their personality. “‘*Confused*’ *sounds bad*,* but it doesn’t always have to be bad. Well*,* it still makes me me*…”, stated Anna. Nora expressed that she sees strengths in her different way of thinking and functioning and sees it as a part of her personality that she has learned to appreciate. Others described that they still are in a process of self-discovery.

Some reflected on their future and how they can find a profession and social context that suits their way of functioning. Some felt safer as an adult because it meant greater freedom to choose contexts. Others thought about how to reconcile their personal and professional identity in the future, Nora said: “*I’ll have to see in future life. But it feels like it’s a bit unpredictable if it will go wrong or not. Do you understand? It would be nice to be myself and understand that it’s not bad to be the way I am now*.”

## Discussion

In the current study, we explored the motives and processes underlying self-diagnosed ADHD in young women, through qualitative interviews and inductive thematic analysis. The results indicate that self-diagnosed ADHD, like a formal diagnosis [18], fulfills an important function in young women’s identity formation through validation of negative experiences and struggles to live up to intrinsic and societal demands. The women recognized themselves in the ADHD diagnosis but felt that their difficulties were not validated by others, and they expressed ambivalence about moving forward with a formal assessment. They mostly displayed the female presentation of ADHD with predominantly inattentive symptoms [33]. Strives to live up to being a “good girl”, including high academic achievement, disguised their experienced difficulties. Their invisible struggles had a negative impact on self-image, and they experienced being left to fend for themselves in dealing with the demands of society, relationships, and striving for self-acceptance.

One of the criteria for ADHD concerns functional impairment, such as academic, social, and/or occupational difficulties [8]. Most respondents reported high functioning coupled with extreme efforts to succeed and survive academically and socially, which in turn negatively affected their well-being. However, psychological suffering is not a criterion for ADHD, nor is the amount of force required to maintain function taken into account. The ambivalence and resistance regarding seeking a formal assessment seemed to stem from a fear of not fulfilling diagnostic criteria regarding functional impairment, which was possibly reinforced by earlier statements and claims by parents and teachers. This meant that the participants found themselves in a kind of *limbo*, experiencing a form of psychological distress while simultaneously believing that they did not meet the criteria for a diagnosis due to their high level of functioning. This underscores the complex and influential social web in which the participants were situated. A web that, to a significant extent, shapes the information we receive about ourselves and the mismatch that this may generate, owing to the narrow roles young women are expected to internalize within the various social contexts [18].

The participants described difficulties with social relationships. They felt different from peers, and described great efforts to adapt to different social contexts by mimicking others, which can be considered a form of camouflaging [44]. This type of camouflaging and identity work resonates with recent research on girls with ADHD [18]. Reasons for the social difficulties were perceived as ADHD symptoms, such as often forgetting about a scheduled meeting or interrupting others. The use of compensatory strategies seems to have helped them to maintain the image of the normative woman and at the same time contribute to the experience of being alone with their difficulties [44]. Of note, the participants belonged to an age group where an adequate approach is to elaborate with identities [28]. Hence, searching for a coherent identity and participating in different social contexts and adapting to social contexts can be regarded as a normative approach for successful identity building. Further, although social camouflaging is highly present in neurodivergent girls, it is also present in neurotypical females, and predictive of anxiety and depression [44].

Society’s norms about what constitutes a successful woman [29, 45] seemed to have reinforced the feeling of being inadequate and different. High academic achievement was an important part of the participants’ identity, and difficulties in dealing with increased academic demands had had a negative impact on self-image. The participants felt that males did not have the same academic expectations, which may have affected the treatment they received in various contexts. The difficulties were dealt with by using compensatory strategies and lowering their own demands.

Unreasonably high demands on women may contribute to medicalization. That is, if the norm is to perform at the top, failures may be attributed to psychiatric diagnoses [46]. ADHD can be considered a popularized diagnosis in Sweden and elsewhere [47] with low perceived stigma [15]. This may contribute to that ADHD can function as an “explanation” for difficulties that may have other origins, such as anxiety, depression, or learning disabilities. Self-diagnosis may in the long run lead to increased requests for assessment even in cases where the difficulties may have other causes, including society’s demands for academic and social achievement or specific gender role expectations [18]. Understanding self-diagnosis in the light of medicalization imply considering the phenomenon in relation to the broader contextual forces that shape the understanding of what is considered “normal”. A continued exploration of the phenomenon of self-diagnosed ADHD should therefore be influenced by this broad societal perspective. However, all participants had self-rated ADHD symptoms above the threshold for probable diagnosis, indicating clinical symptom levels and none was excluded due to low symptom ratings.

The participants described how the self-diagnosis had led to a new understanding of themselves and their difficulties and how it had contributed to their identity development [5]. Similar to a formal diagnosis, self-diagnosis seemed to have offered a form of validation of past experiences and they were able to reformulate their difficulties as an expression of a different functioning rather than personal shortcomings [18, 19, 48]. This allowed them to be more accepting of themselves. In parallel with increased self-understanding, the participants had developed working strategies for dealing with their difficulties and found contexts where they feel that they are allowed to be more of their authentic selves. Their stories testify to an ongoing search for a stable identity. Over time and in parallel with increased self-acceptance, a development towards a more cohesive identity formation takes place [28, 49].

For some participants, identifying with people with ADHD had meant a positive sense of understanding and belonging, something that they express that a formal diagnosis could have strengthened. Others described that the identification had made them feel even more deviant and increased their reluctance to get a formal diagnosis. Thus, self-diagnosis seems to lead to feelings of both social belonging [5] and exclusion in the same way as a formal diagnosis [50]. Studies that have examined the consequences of self-diagnosis in relation to social media and online groups, describe support from those around them as a positive consequence [24]. For the participants in the current study, self-diagnosis did not seem to fulfill this function as they used it for self-understanding rather than understanding from others. This may seem unexpected in light of the frequent media reporting on ADHD influencers and the medicalization of language. One reason for the caution may be that the participants did not consider self-diagnosis as legitimate as a formal diagnosis. Another speculative reason for this caution may have been that the participants functioned relatively well, even though they experienced psychological distress and exhaustion. This may imply that the *problem* was not, in essence, medical but could rather be traced to a societal level, emanating from society’s gendered constructions and expectations: how they should be, what they should do, and how they should perform on the stages of society.

In addition to psychological motives, structural factors can also underly not pursuing a formal diagnosis [5]. Here, participants mention time, energy, and money. Further, despite the fact that reduced stigma around ADHD is seen as a possible explanation for more people seeking assessment [16] and that previous studies have shown that ADHD-related symptoms rather than the actual diagnosis are perceived as stigmatizing [15], the participants in the current study expressed fear of stigmatizing attitudes. However, in line with Hansson Halleröd et colleagues [48], those who considered a formal assessment mentioned access to support and help as a reason for possibly undergoing assessment. As a formal diagnosis is a requisite for receiving medical treatment for ADHD, the self-diagnosed women miss out on this potential intervention.

### Limitations and future research

The sample consisted of relatively high-functioning individuals, which may be unrepresentative of the group of self-diagnosed women. There are probably other groups with self-diagnosed ADHD who have greater difficulties and other experiences of the phenomenon. For instance, we excluded individuals who had high-risk use of alcohol and drugs. Further, participants with great commitment and time to participate may have been overrepresented and the recruitment process through social media may have affected the results.

Further studying perceived differences in self-diagnosis and its impact on identity in men and other age groups is important to further understand the phenomenon, for instance since hormones may be important for symptom presentation in women [51]. Conducting longitudinal studies with the aim of gaining a broader understanding of self-diagnosis and its function over time is another suggestion for future research. Quantifying the results would contribute to increased understanding and generalization of the results. To increase representativeness, cultural background also needs to be studied further. Finally, the issue of why self-diagnosed women may be dissatisfied with receiving (only) affective diagnoses and subsequent treatments as opposed to the self-diagnosed ADHD should be addressed in future studies.

## Conclusions

Self-diagnosis played an important role in the identity development of young women with ADHD-like symptoms. The participants reported significant struggles academically and socially, and they put in a lot of compensatory effort. The sample was academically high functioning, which may explain why perceived difficulties had not been recognized by others. Self-diagnosis seemed to offer validation of negative experiences and facilitated self-understanding. Self-diagnosis had mainly not been used to seek understanding from others and at present they expressed limited need for a formal diagnosis. The results indicate that difficulties in living up to society’s norms for what constitute a successful woman can contribute to self-diagnosis and that this process may contribute to medicalization.

## Supplementary Information

Below is the link to the electronic supplementary material.

Supplementary Material 1

## Data Availability

Availability of data and materials: Due to ethical restrictions, interviews will not be shared in full. Codes and quotes will be shared upon reasonable request.
