# Supplementary material for: Flying under the radar: a qualitative examination of self-diagnosed ADHD and identity formation in young women
Source: BMC Psychol. 2026 Oct 5;14:1435. doi: 10.1186/s40359-026-05732-7 (PMC13637318; doi:10.1186/s40359-026-05732-7)
Supplement: Supplementary file 1 [file 40359_2026_5732_MOESM1_ESM.docx]

Supplementary material

# Interview guide

Could you start by telling us a little about yourself. About who you are and what you like to do in your spare time?

If you think for a moment, is there a difference between who you feel you are and who you would like to be?

Why do you think that you might have ADHD?

(How old were you when you started thinking that it was like that? Have others said anything?)

Why haven’t you done an ADHD assessment?

Have you received any special interventions in school?

Have you received treatment from psychiatry because of this?

Would you have wished that something had been done differently in school or psychiatry?

Will you conduct an assessment in the future? (Why? Why not?)

How do you feel that your schooling and/or study time has been?

Have you during your schooling/studies experienced that you have become someone other than who you want to be or feel that you are?

In what ways has it affected your relationships with classmates and teachers?

How do you feel your working life has been?

Have you experienced at work that you have become someone other than who you want to be or feel that you are? (How have you handled this collision or gap? What do you think could have been done to avoid this?)

In what ways has it affected your relationships with colleagues and managers?

How do you feel that your relationships with other people have been?

Are there experiences that you in relation to others have become someone other than who you want to be or feel that you are?

Are there other contexts that you would like to tell us about where you have experienced similar things, please tell us about this?

Are there relationships where you have experienced that you are allowed to be exactly as you are or become who you want to be?

Now I will ask you some questions about how you feel about being a woman and having ADHD-like symptoms:

Do you feel that the fact that you are a woman has affected how it is and has been for you to live with ADHD-like symptoms?

How would you summarize what self-diagnosed ADHD means to you?

Do you experience any positive aspects with your self-diagnosed ADHD?

Is there any question I should have asked to understand you better?

Is there any question you would like to return to before we finish?
